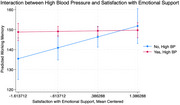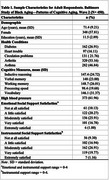# Social Support Satisfaction as a Buffer Against Hypertension‐Related Cognitive Decline in Black Americans

**DOI:** 10.1002/alz70860_107454

**Published:** 2025-12-23

**Authors:** DeAnnah R. Byrd

**Affiliations:** ^1^ Arizona State University, Phoenix, AZ, USA

## Abstract

**Background:**

High blood pressure is a well‐documented risk factor for cognitive decline, yet the role of satisfaction with social support in modifying this relationship remains underexplored, particularly among Black Americans. This study examined whether high blood pressure predicts changes in cognitive functioning over a 3‐year period and whether satisfaction with received emotional or instrumental social support moderates this relationship. Black Americans are disproportionately affected by both hypertension and cognitive decline, making it critical to understand psychosocial factors that may buffer this association.

**Method:**

Data were derived from 450 Black Americans (aged 48–95) in the Baltimore Study of Black Aging—Patterns of Cognitive Aging, with data collection conducted approximately three years apart. Cognitive domains included working memory, processing speed, verbal memory, vocabulary, and inductive reasoning. High blood pressure was determined based on self‐reported diagnosis, with participants asked, “Has a doctor or nurse ever told you that you have/had high blood pressure/hypertension? (0 = no, 1 = yes).” Satisfaction with social support was assessed through participants' ratings of emotional and instrumental support received from family and friends. Data were analyzed using linear regression change models, controlling for covariates.

**Result:**

Sample characteristics are in Table 1. Findings revealed no direct association between high BP and follow‐up cognitive changes. Satisfaction with emotional support marginally (F (3, 433 = 2.30, *p* = 0.0764) moderated this relationship, such that greater satisfaction among those not reporting high BP had higher working memory at follow‐up vs. those with high BP (Figure 1). Conversely, satisfaction with instrumental support did not influence this association across cognitive domains.

**Conclusion:**

These findings underscore the potential of emotional support satisfaction as a modifiable factor that can help mitigate the adverse cognitive effects of high blood pressure, especially among Black Americans, a population at higher risk for hypertension‐related health disparities. Chronic hypertension is associated with vascular damage, reduced brain perfusion, and increased risk of dementia, underscoring the importance of early intervention and targeted protective factors such as social support. Future research should explore mechanisms by which satisfaction with emotional support operates and its implications for designing culturally relevant interventions aimed at preserving cognitive health in this population.